# Prevalence and correlates of multiple non-communicable diseases risk factors among male and female adults in Sudan: results of the first national STEPS survey in 2016

**DOI:** 10.4314/ahs.v22i2.82

**Published:** 2022-06

**Authors:** Supa Pengpid, Karl Peltzer

**Affiliations:** 1 Department of Health Education and Behavioral Sciences, Faculty of Public Health Mahidol University, Bangkok, Thailand; 2 Department of Research Administration and Development, University of Limpopo, Polokwane, South Africa; 3 Department of Psychology, University of the Free State, Bloemfontein, South Africa; 4 Department of Psychology, College of Medical and Health Science, Asia University, Taichung, Taiwan

**Keywords:** Multiple non-communicable diseases, sociodemographic factors, adults, Sudan

## Abstract

**Background:**

Non-communicable diseases (NCDs) are on the rise in low- and middle-income countries. The aim of this study was to assess the prevalence and correlates of multiple NCD risk factors (inadequate fruit and vegetable intake, low physical activity, tobacco use, heavy alcohol use, diabetes, hypertension, raised total cholesterol and overweight/obesity) among adults in Sudan.

**Methods:**

We conducted a cross-sectional study using nationally representative data. The analytic cohort included 7,722 participants who were between the ages of 18–69 years old individuals (median age=36 years) that took part in the “2016 Sudan STEPS survey.”

**Results:**

In all, 34.2% had 0–1 NCD risk factor, 33.5% 2 risk factors, and 32.4% 3 or more NCD risk factors. In adjusted ordinal logistic regression analysis, the odds of having a higher count of NCD risk factors increased from 2.04 to 3.52 from the age group of 35–49 years to age group of 50–69 years when compared to the younger people aged 18–34 years. Men had higher odds (1.21) of higher NCD risk factor count than women. Individuals residing in urban areas had higher odds (1.86) of higher NCD risk factor count than individuals residing in rural areas.

**Conclusion:**

Almost one in three participants had three or more NCD risk factors and several associated variables were identified for men and women that can facilitate in designing intervention programmes.

## Introduction

Non-communicable diseases (NCDs) are estimated to be responsible for 52% of all deaths in Sudan in 2016^1^. More than 85% of NCD premature deaths occur in low- and middle-income countries 2. Cardiovascular diseases, cancers, respiratory diseases, and diabetes contribute to over 80% of all premature NCD deaths 2. Poor diets, tobacco use, harmful alcohol use, and low physical activity all increase the risk of dying from a NCD 2. In the rapid increase of NCDs in sub-Saharan Africa and the Eastern Mediterranean region, it is important to gain insight in the local determinants of NCDs 3,4. Against this backdrop national community-based data on the prevalence of multiple NCD risk factors and associated factors among adults in Sudan are needed, a low-income country geographically in sub-Saharan Africa. Some population-based studies among adults in Sudan were sub-national and only focused on specific NCD risk factors, such as the prevalence of overweight/obesity was 56.1% in four states (Khartoum, Gezira, Blue Nile, and Kassala) 5, 59.0% in Gadarif, Eastern Sudan 6, hypertension was 16.6% in four states (Khartoum, Gezira, Blue Nile, and Kassala) 7, 40.8% in Gadarif, Eastern Sudan 8, the 35.7% in four main cities of the River Nile State, north Sudan 9, 23.6% in the 2005–2006 Khartoum State STEPS survey 10, and 27.6% in Khartoum State in Sudan 11. The prevalence of diabetes was 19.1% in four main cities of the River Nile State, north Sudan 12, 18.7% in the Northern State and River Nile State 13, and 19.8% in the 2005–2006 Khartoum State STEPS survey 10. In a community-based study in Khartoum state, Sudan, the prevalence of physical inactivity was 53.8% 14, the prevalence of current smoking was 12.0% in the 2005–2006 Khartoum State STEPS survey 10, and in a cross-sectional survey of 403 households in Kassala State, Sudan, 72.8% and 36.2% rarely or did not consume fruit and vegetables, respectively 15.

In the 2015 Kenya STEPS survey (18–69 years), 75.8% had 4–12 NCD risk factors (high blood total cholesterol: 10.1%, low blood HDL cholesterol: 57.7%, high sugar intake: 14.9%, inadequate fruit and vegetable intake: 99.8%, obesity: 22.4%, low physical activity: 89.3%, bad fat intake: 39.8%, high intake of salt: 89.5%, hypertension: 24.8%, diabetes: 2.6%, smoking: 10.2%, and harmful alcohol use: 13.8%) 16. In the 2009 Malawi STEPS survey (24–64 years), 16.5% had 3–7 NCD risk factors (raised blood pressure: 32.9%, raised fasting blood glucose: 5.6%, raised cholesterol: 8.7%, overweight or obesity: 26.5%, tobacco smokers: 14.1%, excessive alcohol drinkers: 7.7%, and low physical activity: 9.5%) 17. In the 2014 Uganda STEPS survey (18–69 years), 17.3% had 3–5 NCD risk factor (daily tobacco use, five servings fruit and vegetables, low physical activity, high body mass index and raised blood pressure) 18. In the 2013 Nepal STEPS survey (15–69 years), 27.7% had 3–8 NCD risk factors (current smoking: 18.5%, harmful alcohol use: 2.0%, inadequate fruit and vegetable intake: 98.9%, low physical activity: 3.4%, overweight or obesity: 21.4%, raised blood pressure: 25.7%, raised blood glucose: 3.6%, and raised total cholesterol: 22.6%) 19.

Factors associated with multiple behavioural and biological NCD risk factors include older age 16,19–22, men^19^,^20^, currently married 19, geographic region 18,19, less than higher education 19, higher level of education 20, type of residence 18, better quality of housing 20, higher income level 22, salaried employment 22, and urban residence 20,22. The investigation aimed to estimate the prevalence and correlates of multiple NCD risk factors among 18–69-yearold persons in Sudan.

## Methods

A four-stage stratified cluster sampling method was used to generate representative data of adults aged 18 to 69 years in the cross-sectional Sudan STEPS Survey in 2016, more details of the sample design 23. A total of 7,722 individuals (Median age=36 years; IQR: 23–43) participated in the study. Information on socio-demographic and behavioural NCD risk factors was gathered in Step 1 23. “Physical measurements such as height, weight and blood pressure were collected in Step 2” 23. “Biochemical measurements were collected to assess blood glucose and cholesterol levels in Step 3” 23. Respondents' responses were recorded by the survey administrator on survey tablets (Samsung tablet 4) 23. Blood glucose and cholesterol were measured using cardio-check examination equipment (Cardio check P.A. In vitro diagnostic medical devices for use with PTS panels test strips; Manufacturer: Polymer Technology Systems, INC, Indianapolis, IN USA CE 0197) 23. The response rate for STEP 1 and 2 was 95%, and for STEP 3 88% 23.

### Measures

**Outcome variables:** NCD risk factors were included based on previous studies 16–19, as follows: Behavioural NCD risk behaviour variables included inadequate fruit and vegetable intake (<5 servings/day), low physical activity based on the “Global Physical Activity Questionnaire”, current tobacco use (smoking and/or smokeless tobacco), and episodic heavy alcohol use (six or more in one session) in the past months 23. Biological NCD risk factors. Fasting (≥10 hours) blood sugar measurements were conducted and diabetes was defined as “fasting plasma glucose levels ≥7.0 mmol/L, and/or currently taking insulin or oral hypoglycemic drugs.” 23 Hypertension was assessed based on measured blood pressure (BP) (mean of the last two of three readings) defined as systolic BP ≥140 mm Hg and/or diastolic BP ≥90 mm Hg or currently on antihypertensive medication; raised total cholesterol (TC) (“fasting TC ≥5.0 mmol/L or currently on medication for raised cholesterol”); Body Mass Index (measured 25–29.9kg/m^2^ overweight and ≥30 kg/m^2^ obesity) 23.

Exposure variables included, sex, age, work status, education, household income, residence status and marital status 23.

### Data analysis

Statistical analyses were conducted with STATA software version 15.0 24. To produce representative date for the targeted population, the study sample was “weighted considering the probability of selection at three levels and accounted for participant weight/ individual weight), non-response weight and adjustment for participant's age/sex group (population weight).” 23. The number of NCD risk factors (10) were classified as in previous studies 18,22 into three groups, 1=0–1, 2=2 and 3=3–8 NCD risk factors, and described with frequency counts and bar graphs. Unadjusted and adjusted ordered logistic regression were used to assess predictors of one or more NCD risk factors. Covariates were selected based on previous literature review 16,18–22. Missing values were not included in the analysis. P<0.05 was accepted as significant. Taylor linearization methods were applied to all statistical procedures to account for sample weighting and complex study design.

## Results

### Sample and NCD risk factor characteristics

The sample included 7,722 adults (35.1% males and 64.9% females) aged 18 to 69 years, median age 31 years (IQR: 23–43). About one-third of the participants (34.0%) could not read and write, 51.7% had an household income of 1000 or less Sudanese pounds, and 62.9% were residing in rural areas.

The prevalence of individual NCD risk factors was 94.6% inadequate fruit and vegetable intake, followed by hypertension (31.6%), general overweight/obesity (28.0%), low physical activity (21.3%), current tobacco use (15.7%), raised total cholesterol (13.6%), diabetes (5.9%), and heavy episodic drinking (1.7%). The prevalence of tobacco use and heavy episodic drinking was significantly higher in men than in women, while low physical activity, general overweight/obesity, raised total cholesterol and diabetes was significantly higher in women than in men (Table 1).

**Table 1:** Sample and non-communicable diseases risk factors among 18–69 year-old persons in Sudan, 2016 (N=7722)

Variable	Sample	All	Male	Female	Sex difference
	Unweighted number	%	%	%	p-value
Socio-demographics					
Age in years 18–34 35–49 50–69	3454 2474 1794	57.7 26.5 15.8	58.9 25.3 15.9	56.2 28.0 15.8	0.094
Education Cannot read or write ≤Primary >Primary	3272 2481 1952	34.0 34.2 31.8	24.2 39.7 36.1	45.7 27.6 26.7	<0.001
Household income (Sudanese Pounds) ≤500 501 to ≤1000 1001 to ≤2000 >2000 Do not know	1326 2632 1949 727 1025	18.9 32.8 24.1 10.5 13.6	20.4 33.2 23.7 10.5 12.2	17.2 32.5 24.6 10.5 15.3	0.073
Marital status Never married Married Separated/divorced/widowed	1205 5871 634	30.3 64.8 4.9	40.0 58.4 1.5	18.5 72.4 9.1	<0.001
Employment status Self-employed/non--paid/student/unemployed (able to work) Government employee Non-government employee Homemaker Retired/unemployed (unable to work)	558 858 2389 3677 218	7.9 15.0 43.4 31.4 2.2	9.6 23.1 62.7 1.9 2.6	5.9 5.3 20.0 67.0 1.7	<0.001
Residence Rural Urban	5129 2593	62.9 37.1	62.4 37.6	63.5 36.5	0.563
**Non-communicable diseases** **risk factors**					
Fruit and vegetable intake (<5 servings/day)	410	94.6	95.2	93.9	0.101
Low physical activity	1827	21.3	17.8	25.5	<0.001
Current tobacco use	830	15.7	28.1	0.7	<0.001
Heavy episodic drinking	78	1.7	3.0	0.2	<0.001
Diabetes	515	5.9	5.0	6.8	0.014
Hypertension	2710	31.6	31.1	32.1	0.495
Raised total cholesterol	1229	13.6	8.8	19.5	<0.001
General overweight/obesity	2455	28.0	22.5	35.2	<0.001

### Frequency distribution of multiple NCD risk factors

**Figure 1:**
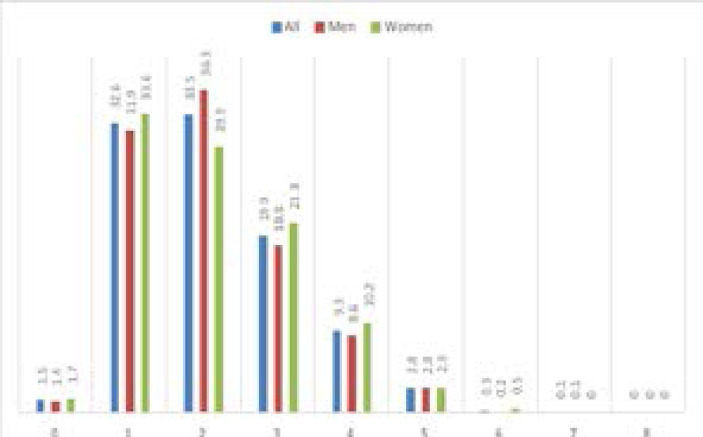
Frequency of non-communicable diseases risk factors among adults in Sudan

The prevalence of having zero NCD risk factors was 1.5%, 1 risk factor 32.6%, 2 risk factors 33.5%, 3 risk factors 19.9%, 4 risk factors 9.3%, 5 risk factors 2.8%, 6 risk factors 0.3%, 7 risk factors 0.1% and 8 risk factors 0% (Figure 1).

Overall, 34.2% had 0–1 NCD risk factor, 33.5% 2 risk factors, 32.4% 3–8 NCD risk factors. Having multiple NCD risk factors incresed with age, urban residence, higher education, higher household income, marital and employment status and sex (Table 2).

**Table 2:** Proportion of multiple non-communicable diseases risk factors (NCDs) among 18–69 year-old persons in Sudan, 2016

Variable	All			Men			Women		
	Number of NCD risk factors			Number of NCD risk factors			Number of NCD risk factors		
	0–1	2	3–8	0–1	2	3–8	0–1	2	3–8
	%	%	%	%	%	%	%	%	%
All	34.2	33.5	32.4	33.3	36.3	30.4	35.3	29.9	34.8
Age (years) 18–34 35–49 50–69	43.8 25.2 15.7	35.7 32.8 26.8	20.4 42.0 57.5	40.8 25.5 17.9	38.2 36.7 28.6	21.0 37.8 53.5	48.1 24.8 13.1	32.3 28.5 24.5	19.6 46.7 62.4
p-value	<0.001			<0.001			<0.001		
Education Cannot read or write ≤Primary >Primary	32.8 36.5 33.2	36.7 32.4 31.3	30.5 31.1 35.5	28.8 35.3 34.2	42.9 34.2 34.0	28.3 30.5 31.8	35.5 38.7 31.6	32.5 29.0 26.5	32.1 32.3 41.9
p-value	0.041			0.103			<0.001		
Marital status Never married Married Separated/divorced/widowed	45.7 29.1 25.8	34.0 34.0 24.0	20.3 36.9 50.2	44.7 25.8 23.7	35.7 36.6 39.1	19.6 37.6 37.2	48.4 32.8 26.3	29.6 31.1 20.8	22.0 36.1 52.9
p-value	<0.001			<0.001			<0.001		
Employment status Self-employed/non- paid/student/unemployed (able to work) Government employee Non-government employee Homemaker Retired/unemployed (unable to work)	24.5 27.0 40.2 33.2 11.5	30.8 36.0 35.5 29.8 30.9	44.7 37.0 24.3 37.0 57.6	22.9 25.0 38.9 38.6 4.1	30.6 37.5 37.0 26.8 31.0	46.5 37.5 24.1 34.5 64.8	27.8 37.6 44.9 33.0 25.7	31.3 28.3 29.7 29.9 30.5	41.0 34.2 25.4 37.1 43.8
p-value	<0.001			<0.001			<0.001		
Residence Rural Urban	38.2 27.4	35.6 29.9	26.1 42.7	36.0 28.7	38.5 32.6	25.5 38.8	41.1 25.8	32.0 26.4	27.0 47.8
p-value	<0.001			<0.001			<0.001		
Household income (Sudanese Pounds) ≤500 501 to ≤1000 1001 ro ≤2000 >2000 Do not know	40.6 36.8 28.0 25.2 37.8	33.8 31.8 34.6 31.4 35.6	25.6 31.3 37.4 43.4 26.6	42.0 36.5 25.2 26.5 32.7	35.6 34.4 37.5 35.4 39.5	22.3 29.1 37.3 38.1 27.9	38.5 37.3 31.5 23.6 43.4	31.0 28.5 31.0 26.6 31.4	30.5 34.2 49.9 25.2 34.9
p-value	<0.001			<0.001			<0.001		

### Associations with multiple NCD risk factors

In adjusted ordinal logistic regression analysis, the odds of having a higher count of NCD risk factors increased from 2.04 to 3.52 from the age group of 35–49 years to age group of 50–69 years when compared to the younger people aged 18–34 years. Men had higher odds (1.21) of higher NCD risk factor count than women. Individuals residing in urban areas had higher odds (1.86) of higher NCD risk factor count than individuals residing in rural areas. Married participants and persons separated, divorced or widowed were also associated with higher odds (1.51 and 1.74, respectively) of possessing higher counts of NCD risk factors. The odds of having a higher count of NCD risk factors increased from 1.61 to 1.75 from the household income of 1001–2000 Sudanese Pounds to the household income of >2000 Sudanese Pounds when compared to those who had a household income of 500 or less Sudanese Pounds. Women who had more than primary education had higher odds (1.38) of higher NCD risk factor count than women who cannot read or write. Among men, the odds of having a higher count of NCD risk factors increased from 1.82 to 3.46 from the government employee group to the retired/unemployed (unable to work) group when compared to the self-employed, engaged in non-paid work, were students or unemployed (able to work) group (Table 3).

**Table 3:** Associations with multiple non-communicable diseases risk factors among 18–69 year-old persons in Sudan, 2016

Variable	All	Men	Women
	AOR (95% CI)	AOR (95% CI)	AOR (95% CI)
Age in years 18–34 35–49 50–69	1 (Reference) 2.04 (1.72, 2.43)*** 3.52 (2.88, 4.31)***	1 (Reference) 1.45 (1.09, 1.93)* 2.26 (1.66, 3.09)***	1 (Reference) 2.73 (2.29, 3.26)*** 5.45 (4.29, 6.94)***
Education Cannot read or write ≤Primary >Primary	1 (Reference) 1.02 (0.85, 1.22) 1.01 (0.81, 1.26)	1 (Reference) 0.97 (0,74, 1.28) 0.84 (0.60, 1.15)	1 (Reference) 1.07 (0.88, 1.30) 1.38 (1.06, 1.80)*
Marital status Never married Married Separated/divorced/widowed	1 (Reference) 1.51 (1.22, 1.87)*** 1.74 (1.22, 2.47)**	1 (Reference) 1.84 (1.34, 2.52)***	1.67 (0.86, 3.26) 1 (Reference) 1.43 (1.11, 1.84)** 1.55 (1.04, 2.29)*
Household income (Sudanese Pounds) ≤500 501 to ≤1000 1001 to ≤2000 >2000 Do not know	1 (Reference) 1.19 (0.95, 1.48) 1.61 (1.26, 2.06)*** 1.75 (1.28, 2.38)*** 1.28 (0.96, 1.71)	1 (Reference) 1.30 (0.95, 1.78) 2.00 (1.41, 2.84)*** 2.01 (1.27, 3.19)** 1.78 (1.24, 2.57)**	1 (Reference) 1.01 (0.79, 1.28) 1.19 (0.91, 1.56) 1.36 (1.01, 1.82)* 0.88 (0.62, 1.24)
Employment status Self-employed/non-paid/ student/unemployed (able to work) Government employee Non-government employee Homemaker Retired/unemployed (unable to work)	1 (Reference) 1.43 (1.06, 1.93)* 1.46 (1.16, 1.83)*** 1.32 (1.08, 1.63)** 2.13 (1.47, 3.07)***	1 (Reference) 1.82 (1.20, 2.77)** 1.59 (1.22, 2.07)*** 1.16 (0.50, 2.73) 3.46 (2.06, 5.83)***	1 (Reference) 0.93 (0.62. 1.42) 1.20 (0.80, 1.80) 1.22 (0.97, 1.54) 1.25 (0.74, 2.11)
Residence Rural Urban	1 (Reference) 1.86 (1.49, 2.32)***	1 (Reference) 1.79 (1.32, 3.41)***	1 (Reference) 1.90 (1.52, 2.37)***

AOR=Adjusted Odds Ratio; CI=Confience Interval; ***p<0.001; **p<0.01; *P<0.05

## Discussion

The present study aimed to assess the prevalence and correlates of multiple NCD risk factors in a national community-based survey among 18–69 year-old individuals in Sudan. In comparison to other low- middle-income countries, the prevalence of 3–8 NCD risk factors (32.4%) in this study (18–69 years) was higher than in the 2009 Malawi STEPS survey (24–64 years) (16.5% 3–7 NCD risk factors) 17, the 2014 Uganda STEPS survey (18–69 years) (17.3% 3–5 NCD risk factors) 18, the 2013 Nepal STEPS survey (15–69 years) (27.7% 3–8 NCD risk factors 19, and lower than in the 2015 Kenya STEPS survey (18–69 years) (75.8% 4–12 NCD risk factors 16. The clustering of three of more NCD risk factors was common in this survey predisposing the adult population in Sudan to a greater risk of NCDs.

In agreement with some studies 16,19–22, this study shows for the first time in a national study in Sudan that older age, male sex, being married, urban residence, higher household income, being in salaried employment, and among women higher education increased the odds for multiple NCD risk factors. Regarding increasing age, early screening, in particular among males, those with higher income, higher education and residing in urban areas, should be propagated to prevent an accumulation of NCD risk factors in Sudan.

The prevalence of hypertension was higher than in previous three local surveys, in four states (Khartoum, Gezira, Blue Nile, and Kassala) (16.6%) 7, in the 2005–2006 Khartoum State SEPS survey (23.6%) 10, and in Khartoum State in Sudan (27.6%) 11, but lower than in two other local surveys in Gadarif, Eastern Sudan (40.8%) 8, and in four main cities of the River Nile State, north Sudan (35.7%) 9. The prevalence of overweight/obesity (28.0%) was lower than in four states (Khartoum, Gezira, Blue Nile, and Kassala) (56.1%) 5, and in Gadarif, Eastern Sudan (59.0%) 6. The high prevalence of inadequate fruit and vegetable intake (94.6%) in this survey was also found in a community in Kassala State, Sudan (72.8% and 36.2% rarely or did not consume fruit and vegetables, respectively) 15. The 2016 Sudan STEPS survey team recommend to “strengthen health literacy and capacity of individuals to make healthy choices e.g. by making fruits and vegetable more affordable” 23.

The prevalence of current tobacco use (15.7%) and heavy episodic alcohol use (1.7%) in this study was in terms of tobacco use similar to the 2005–2006 Khartoum State STEPS survey (current smoking 12.0%) 10, the Kenya STEPS survey (smoking: 10.2%) 16 and the Malawi STEPS survey (tobacco smokers: 14.1%) 17 but lower in terms of heavy drinking in the Kenya STEPS survey (harmful alcohol use: 13.8%) 16 and the Malawi STEPS survey (excessive alcohol drinkers: 7.7%) 17. Increasing exercise taxes and prices on tobacco products may be recommended in Sudan 25.

The prevalence of low physical activity (21.3%) in this survey was lower than in a study in Khartoum state, Sudan, (53.8%) 14. The proportion of raised total cholesterol (13.6%), and diabetes (5.9%) in this study was lower than in the 2005–2006 Khartoum State STEPS survey (raised total cholesterol 19.8% and diabetes 19.8%) 10 in four main cities of the River Nile State, north Sudan (diabetes 19.1%) 12, in the Northern State and River Nile State (diabetes 18.7%) 13, but higher than in the Malawi STEPS survey (raised cholesterol: 8.7% and raised fasting blood glucose: 5.6%) 17 and the Kenya STEPS survey (high blood total cholesterol: 10.1% and diabetes: 2.6% 16.

Some of the found NCD risk factors differed by sex. Current tobacco use and heavy episodic drinking was significantly higher in men than in women, while low physical activity, general overweight/obesity, raised total cholesterol and diabetes was significantly higher in women than in men. In the Kenya STEPS survey daily tobacco and harmful alcohol use was also more prevalent in men than in women, obesity and raised total cholesterol was more common among women than men were 16. Similarly, in the Malawi STEPS survey, the prevalence of alcohol use and tobacco smoking was higher in men than women, and overweight/obesity and raised cholesterol were higher in women than men were 17. The higher prevalence of overweight/obesity in women may be attributed to “in Sudan, obesity is associated with beauty. Furthermore, some young women in Sudan use steroids to gain weight and refuse to take metformin because it is associated with weight loss.” 26.

This study was limited because of the self-report of the interview data as well as its cross-sectional design. Further, the public use dataset of the Sudan 2016 STEPS survey did not include some of the variables, such as region and state, which could therefore not be included in the analysis.

## Conclusion

The study found among a nationally representative population of 18 to 69 years in Sudan that almost one in three participants had three or more NCD risk factors. Several factors associated with an increase in NCD risk count were identified, including older age, male sex, urban residence, higher household income and among women higher level of education, which can assist in guiding interventions to prevent multiple NCD risk factors in the Sudanese population. Considering the clustered nature of NCD risk factors, interventions are needed that target multiple, in particular modifiable, NCD risk factors.

## Data Availability

The data on which this analysis were based are publicly available at the World Health Organization NCD Microdata Repository: URL: https://extranet.who.int/ncdsmicrodata/index.php/catalog.

## Abbreviations

BMI: Body Mass Index; BP: Blood Pressure; HDL: High-density lipoprotein; NCD: Non-communibale diseases; STEPS: STEPwise approach to surveillance; STATA: Statistics and data
